# Elderly Man With Sudden-Onset Abdominal Pain

**DOI:** 10.1016/j.acepjo.2025.100104

**Published:** 2025-03-24

**Authors:** Peter Majoros, Matthias Nuernberger, Jan-Christoph Lewejohann

**Affiliations:** Department of Emergency Medicine, Jena University Hospital, Friedrich Schiller University, Jena, Germany

**Keywords:** acute mesenteric ischemia, abdominal pain, emergency department, aeroportie

## 1 Case Presentation

An 84-year-old man with a history of heart failure, chronic atrial fibrillation on anticoagulation, and previous abdominal surgeries presented to the emergency department with a sudden-onset sharp, stabbing pain in the subdiaphragmatic region, evolving into diffuse abdominal pain. Physical examination revealed a soft abdomen without peritoneal signs, with maximal pain in the left lower quadrant. Initial laboratory tests showed mild lactic acidosis (2.7 mmoL/L), slightly elevated acute-phase proteins (C-reactive protein 15 mg/mL), and leukocytosis (11.6 Gpt/L). Sonography showed no free fluid and no other abnormalities, although the assessment was limited due to the habitus. Analgesics, including opioids, had no effect on the pain.

## 2 Diagnosis and Management of Mesenteric Ischemia

**Figure 1:**
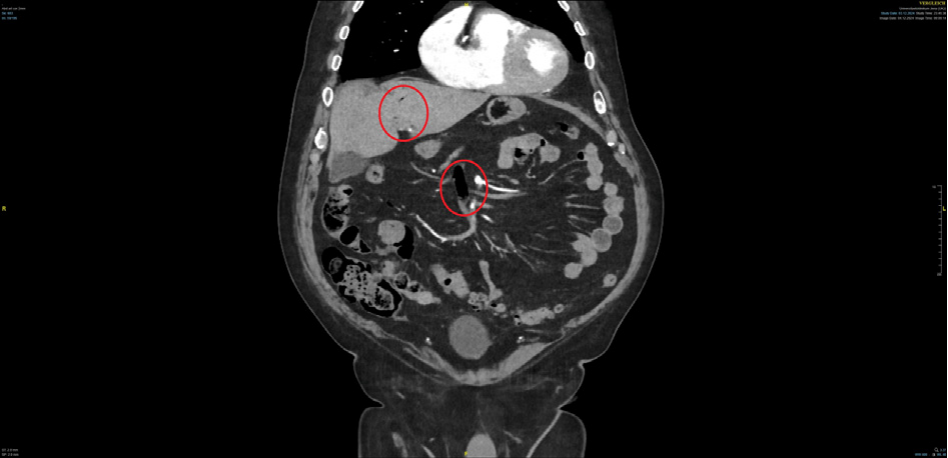
Frontal view of an abdominal angiographic computed tomography scan. Red circles mark gases in the venous system.

**Figure 2:**
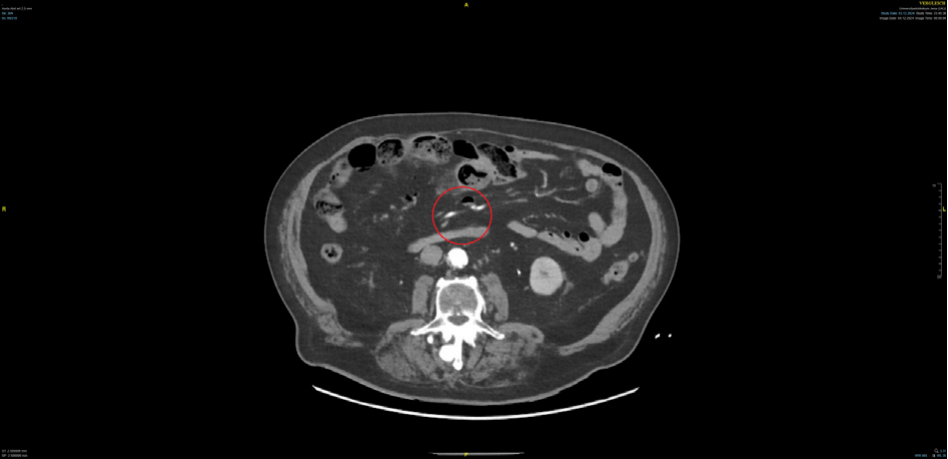
Axial view of an abdominal angiographic computed tomography scan. Red circles mark gases in the venous system.

The persistence of abdominal pain despite analgesia prompted an abdominal and pelvic angiographic computed tomography scan. Imaging revealed gas within the portal venous system, gas within the mesenteric venous system, and a high-grade stenosis of the superior mesenteric artery due to a mixed plaque (see Figures 1 and 2). These findings led to the diagnosis of segmental mesenteric ischemia of the ileum in the right mid-abdomen. Mesenteric ischemia is a critical condition requiring prompt intervention. In this case, the patient’s cardiovascular risk factors and the presence of persistent pain despite adequate analgesia were key clinical indicators. The computed tomography findings were crucial diagnostic features. The patient was immediately transferred for emergency laparotomy and ileal segment resection.

## 3 Teaching Points

This case underscores the importance of maintaining a high index of suspicion for mesenteric ischemia, especially in elderly patients with cardiovascular risk factors. The subtle initial presentation can lead to delayed diagnosis. Persistent abdominal pain despite analgesia, mild lactic acidosis, and inconspicuous sonography should prompt abdominal vascular imaging.

## Conflict of Interest

All authors have affirmed they have no conflicts of interest to declare.

